# Profiling of Differentially Expressed MicroRNAs in Human Umbilical Vein Endothelial Cells Exposed to Hyperglycemia via RNA Sequencing

**DOI:** 10.3390/life13061296

**Published:** 2023-05-31

**Authors:** Nur Syakirah Othman, Amilia Aminuddin, Shahidee Zainal Abidin, Saiful Effendi Syafruddin, Mohd Faizal Ahmad, Norfilza Mohd Mokhtar, Jaya Kumar, Adila A. Hamid, Azizah Ugusman

**Affiliations:** 1 Department of Physiology, Faculty of Medicine, Universiti Kebangsaan Malaysia, Kuala Lumpur 56000, Malaysia; p118667@siswa.ukm.edu.my (N.S.O.); amilia@ppukm.ukm.edu.my (A.A.); norfilza@ppukm.ukm.edu.my (N.M.M.); jayakumar@ukm.edu.my (J.K.); 2 Faculty of Science and Marine Environment, Universiti Malaysia Terengganu, Kuala Nerus 21030, Malaysia; shahidee.zainal@umt.edu.my; 3 UKM Medical Molecular Biology Institute (UMBI), Universiti Kebangsaan Malaysia, Kuala Lumpur 56000, Malaysia; effendisy@ppukm.ukm.edu.my; 4 Department of Obstetrics and Gynaecology, Faculty of Medicine, Universiti Kebangsaan Malaysia, Kuala Lumpur 56000, Malaysia; drmohdfaizal@ukm.edu.my

**Keywords:** apoptosis, diabetes, endothelial dysfunction, human umbilical-vein endothelial cells, hyperglycemia, microRNA, oxidative stress, RNA sequencing

## Abstract

Hyperglycemia is the hallmark of diabetes mellitus that results in oxidative stress, apoptosis, and diabetic vascular endothelial dysfunction. An increasing number of microRNAs (miRNAs) have been found to be involved in the pathogenesis of diabetic vascular complications. However, there is a limited number of studies that characterize the miRNA profile of endothelial cells exposed to hyperglycemia. Therefore, this study aims to analyze the miRNA profile of human umbilical-vein endothelial cells (HUVECs) exposed to hyperglycemia. HUVECs were divided into two groups: the control (treated with 5.5 mM glucose) and hyperglycemia (treated with 33.3 mM glucose) groups. RNA sequencing identified 17 differentially expressed miRNAs between the groups (*p* < 0.05). Of these, 4 miRNAs were upregulated, and 13 miRNAs were downregulated. Two of the most differentially expressed miRNAs (novel miR-1133 and miR-1225) were successfully validated with stem-loop qPCR. Collectively, the findings show that there is a differential expression pattern of miRNAs in HUVEC following exposure to hyperglycemia. These 17 differentially expressed miRNAs are involved in regulating cellular functions and pathways related to oxidative stress and apoptosis that may contribute to diabetic vascular endothelial dysfunction. The findings provide new clues on the role of miRNAs in the development of diabetic vascular endothelial dysfunction, which could be useful in future targeted therapy.

## 1. Introduction

Cardiovascular diseases are one of the major complications of diabetes leading to mortality and morbidity in diabetic patients [1]. Types 1 and 2 diabetes mellitus, despite their different pathogenesis, are characterized by hyperglycemia [2]. Hyperglycemia is responsible for the development of diabetic vascular endothelial dysfunction that eventually leads to the macro- and microvascular complications of diabetes. Endothelial cells are vital for the regulation of vascular homeostasis and are sensitive to changes at the blood glucose level. A growing body of evidence demonstrates that hyperglycemia causes endothelial dysfunction, manifested as increased oxidative stress and apoptosis [2,3].

Endothelial cells exposed to hyperglycemia have increased hydrogen peroxide formation, which promotes oxidative damage to the vasculature [4]. A recent study showed that high glucose exposure for 24 h induces reactive oxygen species (ROS) production and apoptosis in endothelial cells [5]. Hyperglycemia induces the formation of ROS and oxidative stress in diabetes by activating diacylglycerol production, together with the activation of protein kinase C and NADPH-oxidase [6]. ROS accumulation, along with a lack of appropriate adaptation by the endogenous antioxidant defense system, promotes the activation of cellular stress-sensitive pathways that, in turn, contribute to oxidative stress and vascular diabetic complications [7]. 

In addition, oxidative stress causes damage to the lipids, deoxyribonucleic acid (DNA), and proteins in the cells, leading to cellular dysfunction and apoptosis [8]. Hyperglycemia induces apoptosis by activating numerous proteins involved in apoptotic cell death, such as members of the caspase and B-cell lymphoma 2 (Bcl-2) families [9]. Diabetes-induced apoptosis has been observed in the cardiomyocytes and endothelial cells of diabetic patients, and also in streptozotocin-induced diabetic rats and mice [10]. Hence, extensive investigations are now focused on effective strategies to prevent diabetic vascular endothelial dysfunction and its subsequent complications. 

Meanwhile, human umbilical-vein endothelial cells (HUVECs) are a well-established in vitro model of vascular endothelial cell-based research, including those related to diabetes, oxidative stress, and apoptosis [11,12]. HUVECs are also easily accessible and readily available from umbilical cords as discarded biological waste after delivery, rendering them a convenient and abundant source of endothelial cells [13]. 

MicroRNAs (miRNAs) are a family of small (20–24 nucleotides), endogenous, non-coding RNA molecules. A total of 2656 miRNAs have been discovered in the human genome according to the latest version of miRbase database [14]. MiRNAs play diverse roles in fundamental biological processes, including the development of diabetic vascular complications. MiRNAs regulate their target gene expression by binding to a complementary site at the three prime untranslated region (3′ UTR) of the messenger RNA (mRNA), which plays a key role in the conversion of genetic information from gene to proteins [15]. 

Complementary base pairing between a miRNA and its targeted mRNAs of protein-coding genes induces the post-transcriptional repression of the target genes. For example, in the presence of hyperglycemia, miR-134 was highly expressed in human endothelial colony-forming cells. This leads to dysregulated angiogenesis through the inhibition of nuclear receptor-interacting protein-1 activity [16]. Further research found that miR-130a was downregulated in human endothelial progenitor cells (EPC) exposed to hyperglycemia. Consequently, apoptosis of the EPC was increased through the activation of Runt-related transcription factor 3 [16]. Since miRNAs are widely involved in the pathogenesis of diabetes and its complications, it is interesting to explore the effect of hyperglycemia on the profile of miRNAs in vascular endothelial cells, particularly miRNAs that are potentially involved in regulating the oxidative stress and apoptosis pathways in diabetes.

Therefore, this study examines the comprehensive profiling of miRNAs in HUVECs exposed to a hyperglycemic state using RNA sequencing analysis. In addition, we performed bioinformatics analysis to identify key differentially expressed miRNAs, their possible signaling pathways potentially involved in diabetes, oxidative stress, and apoptosis, and a gene ontology (GO) term and Kyoto Encyclopedia of Genes and Genomes (KEGG) enrichment analyses to determine the biological processes and signaling pathways associated with the target genes. Lastly, a protein–protein interaction network was structured on the basis of the STRING database, along with integrated visualization via Cytoscape. Findings from this study may further increase the understanding of miRNAs involved in the development of vascular endothelial dysfunction in diabetes, which can be utilized to further develop targeted therapy.

## 2. Materials and Methods

### 2.1. HUVEC Isolation, Culture, and Treatment

The study was approved by the Universiti Kebangsaan Malaysia research ethics committee (project code: UKM PPI/111/8/JEP-2020-015). All subjects gave their informed consent when they participated in the study. HUVECs were isolated from the umbilical veins by using an enzymatic technique as described previously [17]. Briefly, umbilical cords were cleaned and infused with 0.01% (*w/v*) collagenase (Worthington Biochemical Corporation, Lakewood, NJ, USA). Isolated HUVECs were cultured in an endothelial-cell medium supplemented with 5% fetal bovine serum, 1% endothelial cell growth, and 1% penicillin and streptomycin (ScienCell Research Laboratories, Carlsbad, CA, USA) at 37 °C in a humidified 5% carbon dioxide incubator. The medium was changed every other day until the cells had reached confluence. HUVECs were identified through the typical cobblestone morphology and positive endothelial cell markers (von Willebrand factor and CD31) in immunocytochemistry. 

All the experiments were performed using passages 3–4 of HUVECs at 80% confluency. HUVECs were divided into two groups: the control (treated with 5.5 mmol/L d-glucose) and hyperglycemia (treated with 33.3 mmol/L d-glucose) groups. All treatments were given for 24 h. The dose of glucose and the treatment duration were based on the model of previous studies [18,19,20,21].

### 2.2. Total RNA Extraction

Following 24 h of treatment, total RNA was extracted using the miRNeasy Mini Kit (Qiagen, Hilden, Germany) according to the manufacturer’s instructions. The extracted total RNA was then assessed using an Agilent 2100 Bioanalyzer (Agilent Technologies, Santa Clara, CA, USA), for its integrity, purity, and concentration to guarantee high-quality total RNA for small RNA sequencing.

### 2.3. miRNA Expression Profiling 

The RNA samples were further processed at Genewiz, New Jersey, where the complimentary DNA (cDNA) libraries were prepared and sequenced using Illumina Hiseq, 2 × 150 bp paired-end chemistry. Raw reads were preprocessed for adaptor trimming, quality, and size selection using Trimmomatic (V0.30). Trimmed data were also subjected to quality assessment and assessed using FastQC (V0.10.1). Sequences that passed the quality filtering were mapped to the reference genome, Homo sapiens (GRCh38.104), using Bowtie2 (V2.1.0). The clean reads were aligned to the miRbase database to identify the known miRNAs, and miRDeep2 software was used to predict the novel miRNAs. Raw reads were then normalized to transcripts per million (TPM). Differentially expressed miRNAs were identified using a negative binomial distribution model, available through DESeq2. The generated data in this study were submitted to Gene Expression Omnibus (GEO), accession number GSE229207.

### 2.4. Validation of the Selected miRNAs with Stem-Loop Reverse-Transcription–Quantitative Polymerase Chain Reaction

To validate the RNA sequencing data, two of the most differentially expressed miRNAs (novel miR-1133 and miR-1225) were validated via stem-loop quantitative polymerase chain reaction (qPCR). A total of 5 ng of small RNA-enriched total RNA was synthesized by using an MMLV Reverse Transcriptase 1st-Strand cDNA Synthesis Kit (Biosearch Technologies, Hoddesdon, UK) with an additional 0.1 µM of the stem-loop primer. The qPCR of the novel miR-1133 and miR-1125 was performed in 10 µL of total reaction consisting of 5 μL of 2× miRCURY SYBR Green Master Mix, 0.5 μM of each forward and reverse primer, and 3 μL of the synthesized cDNA (60× diluted). All the primer sequences are listed in Table 1. All reactions were prepared in a 96-well plate format, and qPCR was performed using a CFX96™ Real-Time PCR detection system (Bio-Rad, Hercules, CA, USA). The reactions were conducted using the following thermocycling conditions: initial denaturation at 95 °C for 10 min, followed by 40 cycles of amplification at 95 °C for 10 s, annealing at 60 °C for 30 s, and elongation at 72 °C for 10 s, with an additional elongation step at 40 °C for 1 s. The reaction kinetic of each primer set and protocol was verified with the melting profile. The amplification signals were acquired during the elongation step and recorded by using Bio-Rad CFX Manager software version 3.1. The threshold cycle (C_T_) value was used to calculate the expression of the miRNAs. The U6 gene was used to normalize the quantitative analysis. The relative miRNA expression was calculated with the 2^−∆∆CT^ method, with ∆∆C_T_ = C_T_ of miRNA − C_T_ U6.

### 2.5. Bioinformatics Analysis

GOSeq (v1.34.1) was used to identify GO terms that annotated a list of enriched genes with a significance value of *p* < 0.05. The significant differential gene expression was enriched in KEGG pathways. Following adjustment with Benjamini and Hochberg’s approach for controlling the false discovery rate, the *p*-value of miRNAs was set to <0.05, and the fold change of genes was set to a minimum of a twofold change to detect differentially expressed miRNAs.

### 2.6. Prediction and Analysis of the Potential Target Genes of Differentially Expressed miRNAs

The potential target genes of differentially expressed miRNAs were identified using in silico prediction analysis through independent prediction miRDB algorithms (http://mirdb.org, accessed on 1 March 2023). To date, the only miRNA–mRNA prediction tool available for novel miRNAs is the miRDB algorithm. Since novel miRNAs were part of the differentially expressed miRNAs in this study, the miRDB algorithm was used to predict the target genes and subsequently validate the downstream target of miRNAs.

The predicted target genes in miRDB v6.0 were downloaded using the default parameters with prediction scores ranging from 50 to 100. The predicted target genes of the differentially expressed miRNAs were mapped into the Database for Annotation, Visualization, and Integrated Discovery (DAVID) (v6.8) (https://david.ncifcrf.gov/, accessed on 26 March 2023). The functional annotation clustering of differentially expressed miRNAs was performed against a GO term with medium stringency settings (kappa similarity term overlap of three or more, similarity threshold of 0.50, minimal three group members ab initio with EASE scores of ≥1) to characterize the overall role and function of differentially expressed miRNAs target genes. Fisher’s exact and Chi-square tests were used to determine the significance of the GO terms. Only the GO terms with *p*-value of <0.05 were selected. Then, the protein–protein interaction (PPI) network of target genes was constructed using the STRING database (http://string-db.org, accessed on 28 March 2023). Subsequently, the hub genes in the network were identified by analyzing the degree of connectivity using Cytoscape software (version 3.6.1).

### 2.7. Statistical Analysis

GraphPad Prism version 9 software was utilized to analyze the qPCR data. The Shapiro–Wilk test was used to determine the normality of the data. Results are expressed as mean ± SEM. Differences between the groups were evaluated using unpaired *t*-test. *p*-values < 0.05 were considered statistically significant.

## 3. Results

### 3.1. RNA Sequencing Identified Differentially Expressed miRNAs in Hyperglycemia-Induced HUVECs

**Figure 1:**
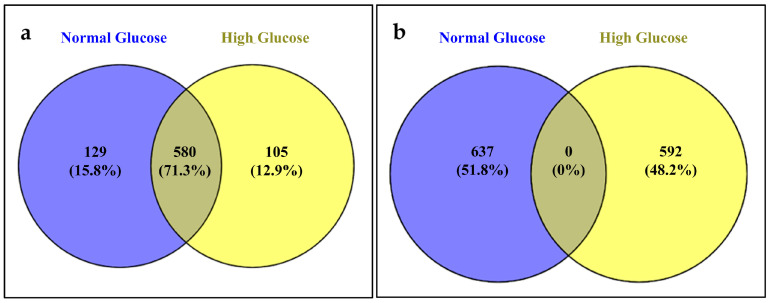
Venn diagram depicting numbers of (**a**) known and (**b**) unknown miRNAs identified in control and hyperglycemia-induced HUVECs.

The deep RNA sequencing of HUVECs following exposure to normal and high glucose for 24 h identified a total of 814 known miRNAs, of which 580 miRNAs were detected in both the control and hyperglycemia groups. We also found a total of 1229 novel miRNAs in these samples (Figure 1). Known miRNAs refer to miRNAs that have been identified and documented in miRNA databases. On the other hand, unknown or novel miRNAs are miRNAs that have not been identified or documented in previous studies or established miRNA databases. However, MiRDeep2 prediction is able to determine possible novel miRNAs on the basis of the secondary structure of miRNA precursors [22].

**Figure 2:**
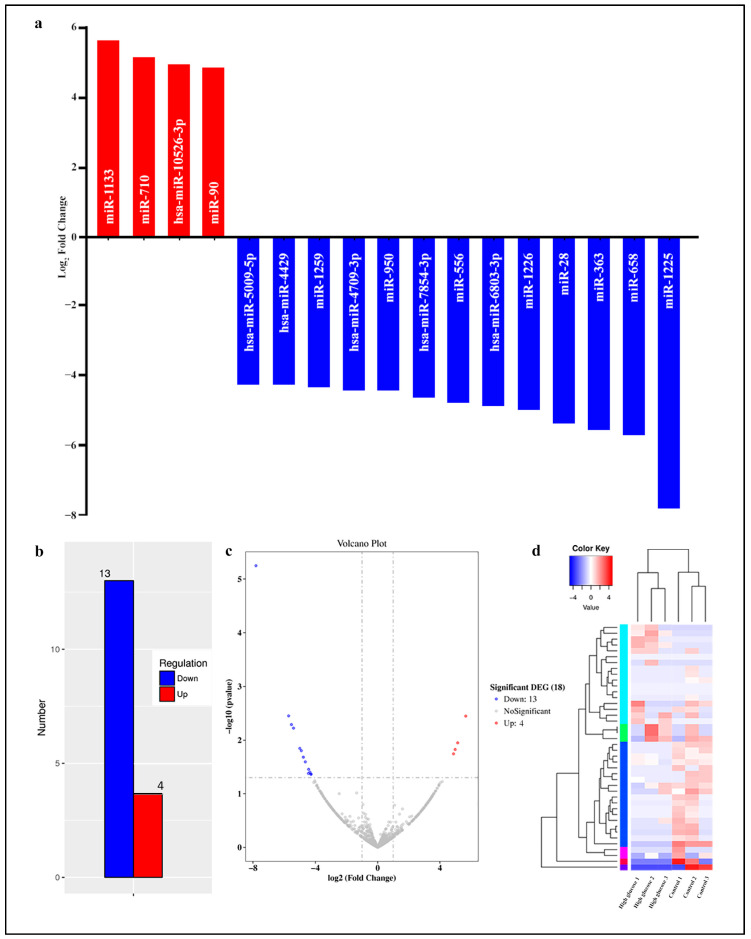
General characterizations of 17 differentially expressed miRNAs in hyperglycemia-induced HUVECs (*n* = 3) (**a**,**b**) Differentially expressed miRNAs displayed in the bar graph of miRNA up- and downregulation across all samples; (**c**) volcano plot. Red and blue dots represent up- and downregulated miRNAs, respectively, with > twofold decrease and increase in expression in hyperglycemia-induced HUVECs, respectively (*p* < 0.05). Gray dots indicate miRNAs with no significant alterations in expression levels. (**d**) Hierarchical clustering analysis of 17 differentially expressed miRNAs in samples using log_2_ transcript per million values. Blue represents miRNAs with low expression and red denotes high expression. Gradient from blue to red represents the increase in miRNA expression.

The patterns of differentially expressed miRNAs were then established between the control and hyperglycemia groups. The DESeq2 analysis of RNA sequencing data distinguished a total of 17 differentially expressed miRNAs (miR-1133, miR-710, has-miR-10526-3p, miR-90, hsa-miR-5009-5p, hsa-miR-4429, miR-1259, hsa-miR-4709-3p, miR-950, hsa-miR-7854-3p, miR-556, hsa-miR-6803-3p, miR-1226, miR-28, miR-363, miR-658 and miR-1225) in the hyperglycemia group compared to the control group (fold change ≥ 2.0, *p* < 0.05) (Table 2). Of these, four miRNAs (miR-1133, miR-710, hsa-miR-10526-3p and miR-90) were upregulated, and 13 miRNAs (hsa-miR-5009-5p, hsa-miR-4429, miR-1259, hsa-miR-4709-3p, miR-950, hsa-miR-7854-3p, miR-556, hsa-miR-6803-3p, miR-1226, miR-28, miR-363, miR-658 and miR-1225) were downregulated. As illustrated in Figure 2, the bar graph of up- and downregulated miRNAs across all samples, volcano plot, and hierarchical clustering analysis revealed that the expression profile of miRNAs in control and hyperglycemia-induced HUVECs were diverse.

### 3.2. Validation of Novel miR-1133 and miR-1225 via Stem-Loop qPCR 

**Figure 3:**
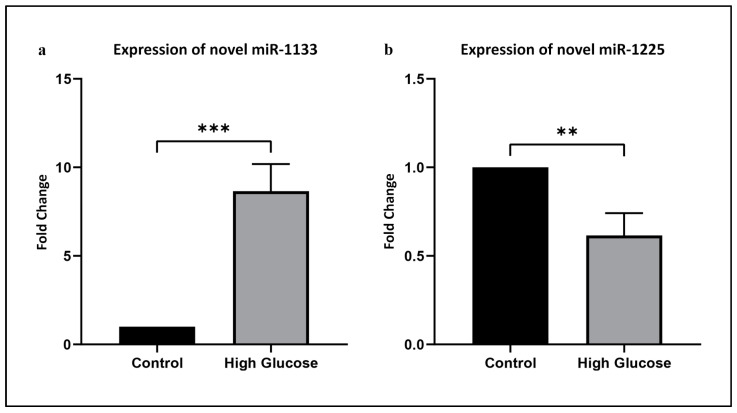
Validation of (**a**) novel miR-1133 and (**b**) novel miR-1225 using stem-loop qPCR. Values are shown as mean ± SEM, *n* = 5 (** *p* < 0.01; *** *p* < 0.001 vs. control).

To validate our RNA sequencing data, we selected one of the most significantly upregulated miRNAs (novel miR-1133) and one of the most significantly downregulated miRNAs (novel miR-1125). These miRNAs were further quantified with stem-loop qPCR. The results confirmed that novel miR-1133 was significantly upregulated by 8.7-fold in HUVECs exposed to hyperglycemia compared to the control HUVECs (*p* < 0.001). Meanwhile, novel miR-1225 was significantly downregulated by 1.62-fold in HUVECs following exposure to hyperglycemia (*p* < 0.01) (Figure 3). 

### 3.3. GO Analysis

**Figure 4:**
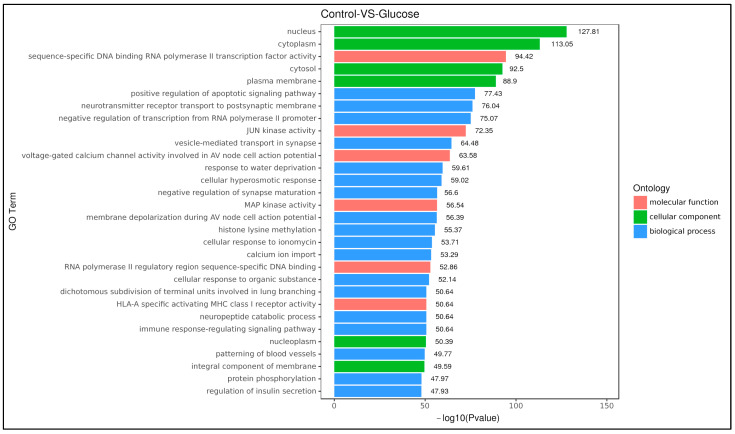
GO functional classification of all differentially expressed miRNAs. GO distributions of the 17 differentially expressed miRNAs in the control vs. hyperglycemia groups were classified into three categories: cellular components (6 subcategories), molecular functions (6 subcategories), and biological processes (18 subcategories).

To better understand the potential implications of the 17 differentially expressed miRNAs, a total of 5655 target genes of the 17 known and unknown, significantly expressed miRNAs were annotated using GO analysis, and their functions were categorized. The GO functional classification of all differentially expressed miRNAs is presented in the histogram and shown as the -log *p*-value with the specification of the relevant biological process, cellular component and molecular function (Figure 4). Among all, 6, 18, and 6 GO terms were significantly involved in molecular function, biological process, and cellular component, respectively, on the basis of the *p*-value. 

The cellular component category was enriched with six GO terms: nucleus, GO:0005634; cytoplasm, GO:0005737; cytosol, GO:0005829; plasma membrane, GO:0005886; nucleoplasm, GO:0005654; integral component of membrane GO:0016021. Under the molecular function category, six GO terms (GO:0061629, GO: 0004705, GO:0086056, GO:0004707, GO:0000977, GO:0032397) were enriched and played a significant role in RNA polymerase II transcription factor, JUN kinase, voltage-gated calcium channel, membrane depolarization during atrioventricular (AV) node cell action potential, MAP kinase, RNA polymerase II transcription regulatory region sequence-specific DNA binding and MHC class 1 receptor activity. 

Among the 18 biological processes involving the target genes of the 17 differentially expressed miRNAs were positive regulation of apoptotic signaling pathway (GO:0043065), neurotransmitter receptor transport to postsynaptic membrane (GO:0098887), negative regulation of transcription from RNA polymerase II promoter (GO:0000122), vesicle-mediated transport in synapse (GO:0016192), response to water deprivation (GO:0009414), cellular hyperosmotic response (GO:0006972), negative regulation of synapse maturation (GO:0050805), membrane depolarization during AV node cell action potential (GO:0086045), histone lysine methylation (GO:0034968), cellular response to ionomycin calcium ion import (GO:0071277), cellular response to organic substance (GO:0071310), dichotomous subdivision of terminal units involved in lung branching (GO:0060448), neuropeptide catabolic process (GO:0010813), immune response-regulating signaling pathway (GO:0002764), patterning of blood vessels (GO:0048514), protein phosphorylation (GO:0006468) and regulation of insulin secretion (GO:0050796).

### 3.4. KEGG Pathway Analysis

**Figure 5:**
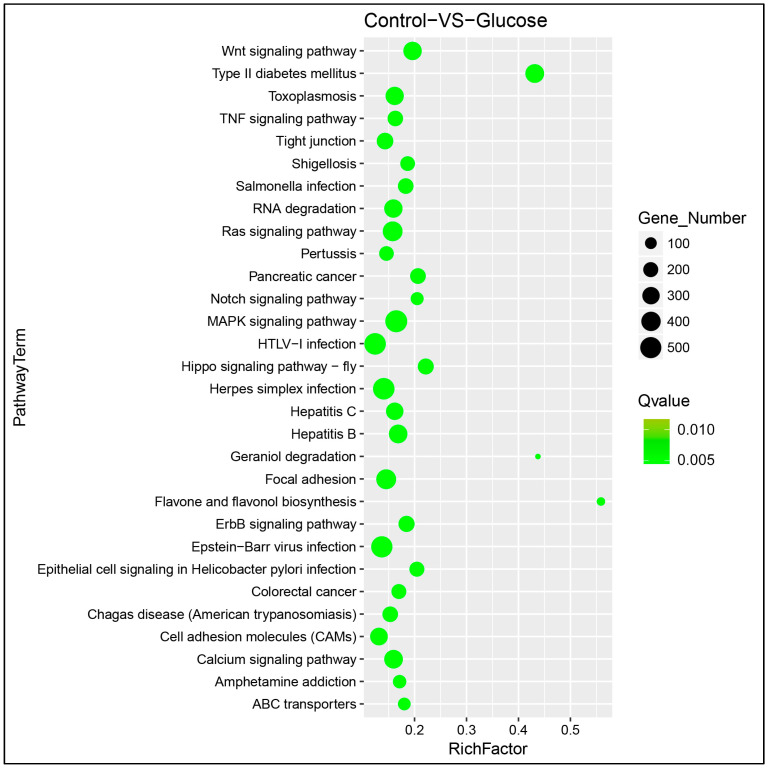
Scatter plot of differentially expressed gene’s KEGG enrichment. The differentially expressed genes were mainly enriched in flavone and flavonol biosynthesis, and Type 2 diabetes mellitus KEGG pathways. The size of the dot is positively correlated with the number of differential microRNA target genes in the pathway. Color code is to indicate different *q*-value ranges. The larger the rich factor is, the greater the degree of enrichment. The smaller the *q*-value is, the more significant the enrichment.

The KEGG pathway database was used to identify diabetes-, oxidative stress-, and apoptosis- related genes involved in the pathways using differentially expressed genes. The the most significant pathways were Type 2 diabetes mellitus, retrograde endocannabinoid signaling, the GnRH signaling pathway, the Wnt signaling pathway, the MAPK signaling pathway, dopaminergic synapse, hippo signaling pathway-fly, insulin secretion, RIG-I-like receptor signaling pathway, prolactin signaling pathway, pancreatic cancer, adipocytokine signaling pathway, hepatitis B, neurotrophin signaling pathway, epithelial cell signaling in *Helicobacter pylori* infection, Ras signaling pathway, ErbB signaling pathway, flavone and flavonol biosynthesis, glycosaminoglycan biosynthesis-keratan sulfate, and calcium signaling pathway with *p*-values between 6.55 × 10^−143^ and 6.10 × 10^−21^ (Figure 5).

### 3.5. Target Prediction and Analysis of Candidate for Differentially Expressed miRNAs

As shown in Table 3, there were a total of 1384 and 6572 predicted targets of the upregulated and downregulated miRNAs, respectively. For the upregulated miRNAs, hsa-miR-10526-3p had the most target genes with the number of 591 target genes. For the downregulated miRNAs, hsa-miR-4429 possessed the most targets of 1047 genes (see Supplementary Materials Table S1 and Figure S1 for details). 

**Figure 6:**
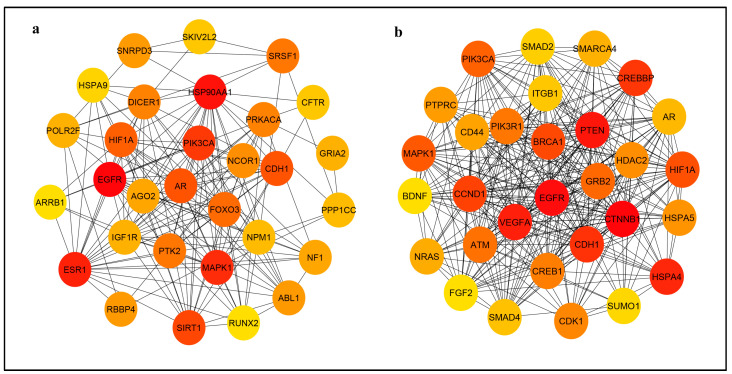
Top 30 hub genes in protein–protein interaction network of the predicted target genes of (**a**) upregulated and (**b**) downregulated miRNAs. Red to yellow CytoHubba nodes represent the highest- to lowest-ranked nodes based on the degree ranking method. ABL1, Abelson murine leukemia viral oncogene homolog 1; AGO2, Argonaut RISC catalytic component 2; AR, androgen receptor; ARRB1, arrestin beta 1; ATM, ataxia telangiectasia mutated; BDNF, brain derived neurotrophic factor; BRCA1, breast cancer gene 1; CCND1, cyclin D1; CD44, CD44 molecule; CDH1, epithelial cadherin; CDK1, cyclin dependent kinase 1; CFTR, cystic fibrosis transmembrane conductance regulator; CREB1, CAMP responsive element binding protein 1; CREBBP, cyclic adenosine monophosphate response element binding protein binding protein; CTNNB1, catenin beta 1; DICER1, dicer 1 ribonuclease III; EGFR, epidermal growth factor receptor; ESR1, estrogen receptor 1; FGF2, fibroblast growth factor 2; FOXO3, forkhead box O3; GRB2, growth factor receptor bound protein 2; GRIA2, glutamate ionotropic receptor AMPA type subunit 2; HDAC2, histone deacetylase 2; HIF1A, hypoxia inducible factor 1 subunit alpha; HSPA4, heat shock protein family A member 4; HSPA5, heat shock protein family A; HSPA9, heat shock protein family A member 9; HSP90AA1, heat shock protein 90 alpha family class A member 1; IGF1R, insulin like growth factor1 receptor; ITGB1, integrin subunit beta 1; MAPK1, mitogen-activated protein kinase 1; NCOR1, nuclear receptor corepressor 1; NF1, neurofibromin 1; NPM1, nucleophosmin 1; NRAS, neuroblastoma ras viral oncogene homolog; PIK3CA, phosphatidylinositol-4,5-bisphosphate 3-kinase catalytic subunit alpha; PI3KR1, phosphatidylinositol 3-kinase regulatory subunit alpha; POLR2F, RNA polymerase II, I and III subunit F; PPP1CC, protein phosphatase PP1-gamma catalytic subunit; PRKACA, protein kinase CAMP-activated catalytic subunit alpha; PTEN, phosphatase and tensin homolog; PTK2, protein tyrosine kinase 2; PTPRC, protein tyrosine phosphatase receptor type C; RBBP4, retinoblastoma binding protein 4; RUNX2, runt-related transcription factor 2; SIRT1, sirtuin 1; SKIV2L2, Superkiller viralicidic activity 2-like 2; SMAD2, mothers against decapentaplegic homolog 2; SMAD4, mothers against decapentaplegic homolog 4; SMARCA4, actin dependent regulator of chromatin subfamily A, member 4; SRSF1, serine and arginine rich splicing factor 1; SUMO1, small ubiquitin like modifier 1; VEGFA, vascular endothelial growth factor A.

Subsequently, the PPI networks of the predicted target genes of four upregulated miRNAs and 13 downregulated miRNAs were separately constructed using the STRING database and Cytoscape software (Figure 6). In the PPI network, it is crucial to find the hub genes that influence oxidative stress in diabetes. Hub genes refer to genes that exhibit a high degree of connectivity or interaction with other genes within the network [23]. According to the degree, the top 12 hub genes in the networks were screened and are listed in Table 4. For the upregulated miRNAs, the top 12 hub genes were epidermal growth factor receptor (EGFR), heat shock protein 90 alpha family class A member 1 (HSP90AA1), estrogen receptor 1 (ESR1), mitogen-activated protein kinase 1 (MAPK1), phosphatidylinositol-4,5-bisphosphate 3-kinase catalytic subunit alpha (PIK3CA), sirtuin 1 (SIRT1), androgen receptor (AR), epithelial cadherin (CDH1), hypoxia inducible factor 1 subunit alpha (HIF1A), forkhead box O3 (FOXO3), serine and arginine rich splicing factor 1 (SRSF1) and protein tyrosine kinase 2 (PTK2). For the downregulated miRNAs, the top 12 hub genes were catenin beta 1 (CTNNB1), EGFR, phosphatase and tensin homolog (PTEN), vascular endothelial growth factor A (VEGFA), heat shock protein family A member 4 (HSPA4), CDH1, cyclic adenosine monophosphate response element binding protein binding protein (CREBBP), cyclin D1 (CCND1), breast cancer gene 1 (BRCA1), HIF1A, MAPK1 and PIK3CA. EGFR showed the highest node degree in both sets, which were 121 and 487, respectively (see Supplementary Materials Tables S2 and S3 for details). 

## 4. Discussion

Hyperglycemia is the hallmark of diabetes mellitus that results in oxidative stress, with subsequent damage to cellular components and apoptosis [24]. The role of miRNAs has been reported in the pathogenesis of various diseases including diabetes mellitus. In this study, we conducted a comprehensive profiling of 17 differentially expressed miRNAs in HUVECs exposed to hyperglycemic state, which is a hallmark of diabetes. Our subsequent integrated bioinformatic analyses identified potential pathways associated with hyperglycemia-induced endothelial oxidative stress. 

RNA sequencing revealed that a number of miRNAs were present in both hyperglycemia-induced and control HUVECs. A total of 17 differentially expressed miRNAs were found comprising 13 downregulated and 4 upregulated miRNAs. We subsequently verified the expression of the most upregulated and downregulated miRNAs in the samples: novel miR-1133 and miR-1125 by stem-loop qPCR. The stem-loop qPCR results of novel miR-1133 and miR-1125 expression were in agreement with the results of high-throughput sequencing, which indicates that the high-throughput RNA-sequencing for screening the differentially expressed miRNAs in our study was accurate and reliable.

We then assessed the GO and pathway enrichment analysis that showed the functions and pathways targeted by the 17 differentially expressed miRNAs. In total, we discovered 3992 notable GO terms and 308 pathways. The wide target range suggests that these miRNAs may play pivotal roles in the biological processes of HUVECs in hyperglycemic state. The functions of the 17 differentially expressed miRNAs involve the regulation of apoptotic signaling pathway, response to water deprivation, cellular hyperosmotic response, histone lysine methylation, cellular response to organic substance, neuropeptide catabolic process, immune response-regulating signaling pathway, protein phosphorylation, and regulation of insulin secretion. Most of these functions are closely related to oxidative stress and apoptosis, and could significantly contribute to the pathogenesis of diabetic vascular complications. In addition, the miRNAs targeted multiple signaling pathways. One of the most enriched KEGG pathways of the 17 differentially expressed miRNAs was the Type 2 diabetes mellitus pathway, which is related to the study. 

miRNA–mRNA network analysis connects the 17 differentially expressed miRNAs to their predicted target genes and the related biological functions, allowing for a deeper understanding of the critical role of dysregulated miRNAs. In the networks, certain miRNAs function as network hubs, such as hsa-miR-10526-3p, mir-90 and miR-70 (upregulated miRNAs); and hsa-miR-4429, miR-28 and hsa-miR-4709-3p (downregulated miRNAs). Of these miRNAs, hsa-miR-10526-3p and hsa-miR-4429 were the most active miRNAs in the networks, targeting 591 and 1047 genes, respectively. Only one study has investigated hsa-miR-10526-3p. In silico analysis has shown that hsa-miR-10526-3p is 1 of the 14 human miRNAs with the highest number of nucleotide matches with the nucleocapsid region of SARS-CoV-2. Thus, inhibiting these miRNAs is a potential way to treat SARS-CoV-2 infection [25]. However, hsa-miR-10526-3p had not been reported to be involved in oxidative stress and apoptosis. 

Nevertheless, hsa-miR-4429 has attracted much attention in recent years. A study showed that cadmium exposure downregulates hsa-miR-4429 in adenocarcinomic human alveolar basal epithelial cells [26]. Cadmium is a potent cell poison that induces oxidative stress through the generation of ROS and by depleting the intrinsic antioxidant reserves [26]. Another study revealed that advanced glycation end products downregulate hsa-miR-4429 to promote apoptosis and fibrosis in pelvic organ prolapse [27]. Moreover, circulating hsa-miR-4429 levels were reduced following ischemic stroke [27], and hsa-miR-4429 was downregulated in the retinal pigment epithelial cells of diabetic mice [28]. Although the specific mechanisms of function of hsa-miR-4429 are not well-understood, findings from previous studies demonstrated that the downregulation of has-miR-4429 exerts a modulatory effect on oxidative stress and apoptosis. Therefore, further studies are needed to elucidate the precise role hashsa-miR-10526-3hasnd hsa-miR-4429 in hyperglycemia-induced endothelial oxidative stress.

In the PPI network of predicted target genes, we found that EGFR had the highest overlap degree of interaction in both upregulated and downregulated miRNAs. EGFR is critically involved in regulating a variety of cellular functions, such as cell growth, proliferation, motility, and survival [29]. The activation of EGFR signaling has been implicated in numerous pathophysiological processes, including diabetes [30]. Emerging studies reported that EGFR can play a dual role. In physiological conditions, EGFR acts as a beneficial factor for the normal development and function of the cardiovascular system. However, when its regulation is disrupted, EGFR acts as a detrimental factor that contributes to cardiovascular pathologies. EGFR inhibition reduced systemic oxidative stress in diabetic mice [30]. Hyperglycemia also increases the ROS that disturbs the EGFR pathway, resulting in delayed corneal epithelial wound healing [31]. While it may not be surprising to see ubiquitous hub genes such as EGFR among the highly ranked genes in both upregulated and downregulated gene lists, it is important to consider their potential functional implications. The fact that EGFR is highly ranked in both lists suggests its involvement in multiple regulatory mechanisms or pathways affected by hyperglycemia. Hence, further study is required to validate the function of significant miRNAs on their target genes such as EGFR.

PI3K acts as an effector of EGFR in diabetes-induced vascular dysfunction [30]. We identified the PI3KCA gene among the highest degrees of PPI network interaction in both upregulated and downregulated miRNAs. PI3K/AKT signaling is a common target downstream of epidermal growth factor receptor/epidermal growth factor receptor family (EGFR/ErbB) signaling. This pathway can activate endothelial nitric oxide synthase to produce nitric oxide (NO), which plays an important role in regulating endothelial function. Impaired PI3K/AKT signaling pathway leads to endothelial dysfunction in diabetic mice as evidenced by the reduction in vasorelaxation and NO production [32]. The PI3K–Akt signaling pathway is also involved in enhancing insulin sensitivity [33]. Moreover, PI3K expression was decreased in oxidative stress-induced HUVECs [34]. Thus, further research is required to validate these target genes that are involved in hyperglycemia-induced oxidative stress in endothelial cells.

Although we performed a comprehensive analysis on the miRNA profile of HUVECs exposed to hyperglycemia, there are a few limitations in this study. First, the sample of the RNA sequencing was relatively small with three samples, hindering identifying less predominantly differentiated miRNAs due to the low statistical power. Second, despite the high similarity of miRNA expression in endothelial cells isolated from various locations, some diversity of the miRNA profile could be present among the different types of endothelial cells [35]. Nevertheless, the scope of this study was limited to the miRNA profile of hyperglycemia-induced oxidative stress in HUVECs. In order to identify the groups of miRNAs that cause vascular complications in a particular target organ, it is necessary to conduct further research on the endothelial cells derived from the blood vessels of the target organ of interest. Lastly, this study was limited by the absence of biological validation to confirm the functional impact of the significant miRNAs, including their effects on the target genes such as EGFR, hence needing further functional study in the future. 

## 5. Conclusions

Collectively, this study showed that 17 miRNAs are differentially expressed in HUVECs following exposure to hyperglycemia. Bioinformatic analyses predict the functions, signaling pathways, target genes, miRNA–gene, and protein networks related to oxidative stress and apoptosis, which may contribute to diabetic vascular endothelial dysfunction. The findings provide new clues on the role of miRNA in the development of diabetic vascular endothelial dysfunction, which could be useful in future targeted therapy. Further functional study is needed to validate the role of these miRNAs in regulating oxidative stress and apoptosis in endothelial cells exposed to hyperglycemia.

## Figures and Tables

**Table 1:** Primer sequence for qPCR.

Target miRNA	Primer Sequence (5′-3′)		PCR Product Size (bp)
1133	Forward	GCTGGGCGGCTTGCTGG	72
	Reverse	GTAGGATGCCGCTCTCAG	
1125	Forward	GATCTGCTGCAGTGCTC	72
	Reverse	GTAGGATGCCGCTCTCAG	
U6	Forward	CTCGCTTCGGCAGCACA	94
	Reverse	AACGCTTCACGAATTTGCGT	
Stem-loop miR-1133	GTTGGCTCTGGTAGGATGCCGCTCTCAGGGCATCCTACCAGAGCCAAACCGAGCC		
Stem-loop miR-1225	GTTGGCTCTGGTAGGATGCCGCTCTCAGGGCATCCTACCAGAGCCAAACGGCTCA		

**Table 2:** Differentially expressed microRNAs (miRNAs) between the control and hyperglycemia groups (*p* < 0.05, fold change ≥2).

miRNA	Fold Change	Type of Regulation	*p*-Value
Novel miRNA-1133	5.656155105	Ups	0.003556
Novel miRNA-710	5.138058047	Ups	0.011192
hsa-miR-10526-3p	4.97619696	Ups	0.014895
Novel miRNA-90	4.867705436	Ups	0.017918
hsa-miR-5009-5p	−4.27746075	Down	0.043118
hsa-miR-4429	−4.27746075	Down	0.043118
Novel miRNA-1259	−4.342495533	Down	0.039524
hsa-miR-4709-3p	−4.43050579	Down	0.034903
Novel miRNA-950	−4.445521443	Down	0.041609
hsa-miR-7854-3p	−4.64142907	Down	0.025308
Novel miRNA-556	−4.769735545	Down	0.020703
hsa-miR-6803-3p	−4.897376412	Down	0.015674
NovelmiRNA-1226	−4.995100139	Down	0.0142
Novel miRNA-28	−5.397699054	Down	0.00595
Novel miRNA-363	−5.535195224	Down	0.005111
Novel miRNA-658	−5.715530062	Down	0.003519
Novel miRNA-1225	−7.810394044	Down	5.63 × 10^−6^

**Table 3:** Target number of differentially expressed miRNAs.

Upregulated miRNA	Number	Downregulated miRNA	Number
hsa-miR-10526-3p	591	hsa-miR-4429	1047
miR-90	447	miR-28	769
miR-710	214	hsa-miR-4709-3p	759
miR-1133	132	miR-1226	668
		hsa-miR-7854-3p	596
		miR-1259	593
		miR-556	551
		miR-363	524
		miR-1225	381
		hsa-miR-5009-5p	338
		miR-950	263
		miR-658	45
		hsa-miR-6803-3p	38
Total	1384	Total	6572

**Table 4:** Hub genes identified in the protein–protein interaction networks.

Upregulated miRNAs		Downregulated miRNAs	
Gene Symbol	Degree	Gene Symbol	Degree
EGFR	121	CTNNB1	487
HSP90AA1	110	EGFR	422
ESR1	98	PTEN	320
MAPK1	79	VEGFA	263
PIK3CA	72	HSPA4	259
SIRT1	71	CDH1	255
AR	64	CREBBP	253
CDH1	64	CCND1	245
HIF1A	61	BRCA1	241
FOXO3	57	HIF1A	238
SRSF1	56	MAPK1	224
PTK2	56	PIK3CA	222

AR, androgen receptor; BRCA1, breast cancer gene 1; CCND1, cyclin D1; CDH1, epithelial cadherin; CREBBP, cyclic adenosine monophosphate response element binding protein binding protein; CTNNB1, catenin beta 1; EGFR, epidermal growth factor receptor; ESR1, estrogen receptor 1; FOXO3, forkhead box O3; HIF1A, hypoxia inducible factor 1 subunit alpha; HSPA4, heat shock protein family A member 4; HSP90AA1, heat shock protein 90 alpha family class A member 1; MAPK1, mitogen-activated protein kinase 1; PIK3CA, phosphatidylinositol-4,5-bisphosphate 3-kinase catalytic subunit alpha; PTEN, phosphatase and tensin homolog; PTK2, protein tyrosine kinase 2; SIRT1, sirtuin 1; SRSF1, serine and arginine rich splicing factor 1; VEGFA, vascular endothelial growth factor A.

## Data Availability

Publicly available datasets were analyzed in this study. These data can be found in GEO, accession number GSE229207.

## Supplementary Materials

The following supporting information can be downloaded at: https://www.mdpi.com/article/10.3390/life13061296/s1. Supplementary Table S1: list of target genes for differentially expressed miRNAs. Supplementary Figure S1: predicted target genes of (a) upregulated and (b) downregulated miRNAs, highlighting the presence of a higher number of connected genes. Supplementary Table S2: top 30 upregulated genes in network STRING_interactions. Supplementary Table S3: top 30 downregulated genes in network STRING_interactions.
