# Supplementary figures and images for: Profiling of Differentially Expressed MicroRNAs in Human Umbilical Vein Endothelial Cells Exposed to Hyperglycemia via RNA Sequencing
Source: Life (Basel). 2023 May 31;13(6):1296. doi: 10.3390/life13061296 (PMC10304839; doi:10.3390/life13061296)

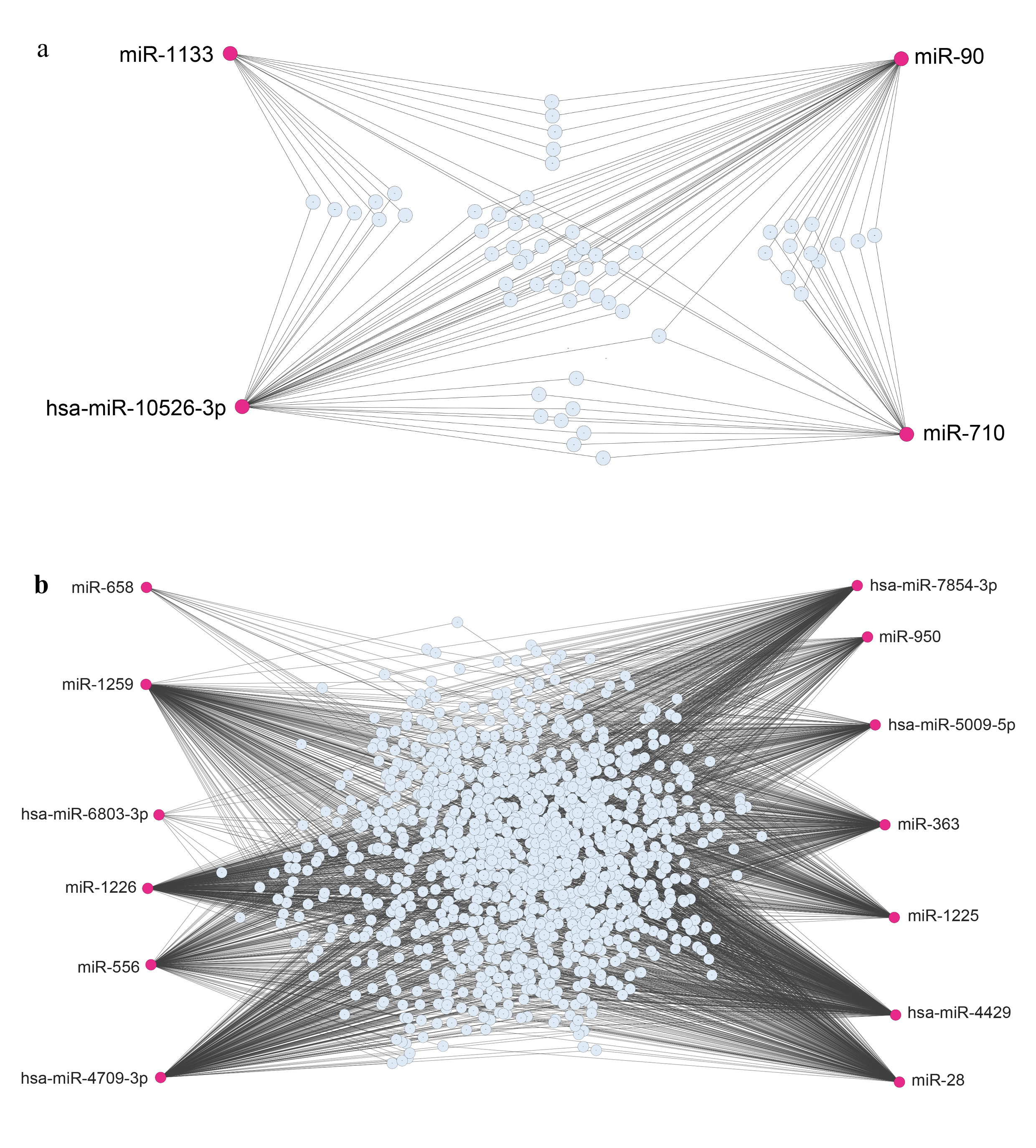

Supplement: Supplementary file 1 [file life-13-01296-s001.zip › Supplementary Figure S1 Predicted target genes of the upregulated (a) and downregulated (b) miRNAs, highlighting the presence of a higher number of connected genes..tif]
